# Effect of the type of brewing water on the sensory and physicochemical properties of light-scented and strong-scented *Tieguanyin* oolong teas

**DOI:** 10.1016/j.fochx.2023.101099

**Published:** 2023-12-23

**Authors:** Yuan-Yuan Ma, Jie-Qiong Wang, Ying Gao, Qing-Qing Cao, Fang Wang, Jian-Xin Chen, Zhi-Hui Feng, Jun-Feng Yin, Yong-Quan Xu

**Affiliations:** a Tea Research Institute, Chinese Academy of Agricultural Sciences, Key Laboratory of Biology, Genetics and Breeding of Special Economic Animals and Plants, Ministry of Agriculture and Rural Affairs, 9 South Meiling Road, Hangzhou 310008, China; b Graduate School of Chinese Academy of Agricultural Sciences, Beijing 100081, China

**Keywords:** (+)-Catechin (PubChem CID: 1203), (−)-Epigallocatechin gallate (PubChem CID: 65064), (−)-Epicatechin gallate (PubChem CID: 367141), (−)-Epigallocatechin (PubChem CID: 72277), (−)-Epicatechin (PubChem CID: 72276), (−)-Gallocatechin gallate (PubChem CID: 199472), (−)-Catechin gallate (PubChem CID: 6419835), (−)-Gallocatechin (PubChem CID: 9882981), Brewing water, *Tieguanyin* oolong tea, Taste-contributing metabolites, Volatile components

## Abstract

Variations in the quality of brewing water profoundly impact tea flavor. This study systematically investigated the effects of four common water sources, including pure water (PW), mountain spring water (MSW), mineral water (MW) and natural water (NW) on the flavor of *Tieguanyin* tea infusion. Brewing with MW resulted in a flat taste and turbid aroma, mainly due to the low leaching of tea flavor components and complex interactions with mineral ions (mainly Ca^2+^, Mg^2+^). Tea infusions brewed with NW exhibited the highest relative contents of total volatile compounds, while those brewed with PW had the lowest. NW and MSW, with moderate mineralization, were conducive to improving the aroma quality of tea infusion and were more suitable for brewing both aroma types of *Tieguanyin*. These findings offer valuable insights into the effect of brewing water on the sensory and physicochemical properties of oolong teas.

## Highlights

• Flavonoids, organic acids concentrations were greatly affected by brewing water.
• Water ions greatly impact the purity and richness of tea infusions’ aroma.
• Moderate mineralization improves the aroma quality of *Tieguanyin* tea infusion.

## 1 Introduction

Tea is one of the most popular and widely consumed beverages worldwide, renowned for its diverse flavors and health benefits (Yang et al., 2023a). Brewing, the final step before tea consumption, has garnered extensive attention due to its close association with the sensory quality and physicochemical properties of tea infusion. The leaching of various tea components, related to flavor or bioactivity, can be significantly affected by brewing factors, such as water, temperature, and brewing time, resulting in the differences in flavor and even health-related properties (Deng et al., 2023, Tang et al., 2016, Yu et al., 2021). Among these factors, the quality of brewing water, especially its pH and mineral content, is of utmost concern. The mineral concentration and pH value of water significantly influence the contents of volatile compounds, and the water with neutral pH and lower mineral content is more conducive for brewing high-quality green tea (Bai et al., 2023). Mineral-rich water adversely affects the flavor of the tea infusion, as well as the concentration of catechins and the antioxidant capacity of the tea infusion (Xu et al., 2017). A previous study has shown that brewing water containing high concentrations of Ca^2+^ can diminish desirable tastes and increase astringency of tea infusions (Xu et al., 2017). Additionally, Ca^2+^ has a significant effect on the aroma of green tea, reducing its freshness and resulting in a stuffy sensory perception (Liu et al., 2016). Furthermore, the contents of volatile components of green tea infusion are also correlated with the concentrations of Mg^2+^ and the pH of water (Liu et al., 2016).

Among the various types of tea, oolong tea stands out for its characteristic flavor and bioactivity, which results from the unique processing step of semi-fermentation (Wang et al., 2022, Zeng et al., 2022a). *Tieguanyin* oolong tea, originating from Fujian province in China, holds particular esteem for its distinctive aroma profiles. Its quality depends on the cultivar, growing conditions, and processing techniques. *Tieguanyin* oolong tea is divided into light-scented and strong-scented types according to the aroma (Cao et al., 2021a). The light-scented *Tieguanyin* (LST) undergoes light rolling, fermentation, and roasting, presenting a fresh and floral aroma. In contrast, the processing of strong-scented *Tieguanyin* (SST) involves a longer period of rolling and fermentation, followed by dark roasting, resulting in a strong and sweet floral aroma. The flavor of *Tieguanyin* tea distinguishes it from green and black teas, but there isn’t focused investigation yet to the influence of brewing water on the its’ sensory and physicochemical characteristics.

In this study, we selected four commonly used brewing waters with different mineral contents, including pure water (PW), mountain spring water (MSW), natural water (NW), and mineral water (MW) based on previous studies (Cao et al., 2021b, Li et al., 2023). The effects of four water on the flavor attributes as well as the composition of non-volatile and volatile components of brewed *Teiguanyin* tea infusion were investigated using a multi-platform instrumental analysis. The findings can be utilized to improve the overall sensory quality of both LST and SST infusions by choosing the optimal brewing water. It also provides insights for the development of high-quality tea brewing water selection based on flavor harmonization.

## 2 Materials and methods

### 2.1 Materials

#### 2.1.1 Tea and water samples

The tea samples (LST and SST; special grade), obtained from the Anxi County Yunling Tea Industry Co., Ltd (Anxi, China), were processed from the same batch materials according to the traditional manufacturing technology of *Tieguanyin* oolong tea, which includes withering, shaking, fixation, rolling, baking and drying. Four experimental brewing water samples were pure water (PW) from Hangzhou Wahaha Group Co., Ltd. (Hangzhou, China), mountain spring water (MSW) from Hangzhou Tongsheng Water Co., Ltd (Hangzhou, China), mineral water (MW) from Natural Waters of Viti Co., Ltd. (Lautoka, Fiji), and natural water (NW) from Nongfu Spring Co., Ltd. (Hangzhou, China).

#### 2.1.2 Chemicals

Acetonitrile, formic acid, and methanol were all HPLC grade and from Merck (Darmstadt, Germany). Authentic standards were purchased from Sigma–Aldrich (Shanghai, China), including caffeine, gallic acid, (+)-Catechin (C), (−)-epicatechin (EC), (−)-gallocatechin (GC), (−)-epigallocatechin (EGC), (−)-catechin gallate (CG), (−)-epicatechin gallate (ECG), (−)-gallocatechin gallate (GCG), (−)-epigallocatechin gallate (EGCG). Acetic acid was purchased from Aladdin Co., Ltd (Shanghai, China). Ethyl caprate (decanoate; 99 %) and *n*-alkanes (C7-C40) were purchased from Beijing Zhongsheng Ruitai Technology Co., Ltd. (Beijing, China).

### 2.2 Preparation of tea infusions

Tea leaves were brewed with four different boiling waters (PW, MSW, MW and NW) at a leaf/water ratio of 1:50 (*w*/*v*) for 6 min (Gong et al., 2018). Tea infusions were obtained by filtering to remove the tea leaves and immediately cooled to room temperature (RT, 25 ± 2 °C) in a cooling tank. Each preparation of tea infusion was repeated three times on different days and the same went for the below experiments. Subsequently, sensory evaluation and physicochemical analysis were performed to investigate the effects of different types of water on the sensory characteristics and chemical composition of the tea infusions.

### 2.3 Analysis of physicochemical properties of water samples

The physicochemical properties (including pH, conductivity, and the concentrations of anions and metal cations) of the four water samples were analyzed. The pH values were measured using a pH meter (FiveGo F2 pH Meter; Mettler Toledo Instruments, Shanghai, China) after calibrating with buffer solutions of pH 4.01, 6.86, and 9.18 (Mettler-Tolrdo). The conductivity was determined using a conductivity meter (HQ30d Flexi Meter, HACH Company, Loveland, CO) (Cao et al., 2021b). Besides, the concentrations of the anions in water were determined by ion chromatography (Dionex, Thermo Fisher Scientific Inc. Shanghai, China) (Xu et al., 2017). They were analyzed after calibration with a standard solution containing 5 mg/L each of HCO_3_^−^, Cl^−^, NO_3_^−^, and F^−^. The metal cation composition (Ca^2+^, K^+^, Mg^2+^, and Na^+^) of the brewing water was analyzed by inductively coupled plasma mass spectrometry (ICP-MS; Thermo Jarrell Ash Corp. USA). The analytical conditions were as follows: a charge injection device detector; low wavelength maximum integration time: 15 s; high wavelength maximum integration time: 5 s; nebulizer pressure: 28 psi; pump speed: 100 rpm; auxiliary gas flow: 1 L/ min; and RF power: 1150 W.

### 2.4 Sensory evaluation of tea infusions

The sensory quality of tea infusions was evaluated by six trained panelists (two males, four females, aged 25–48) from the Tea Research Institute of Chinese Academy of Agricultural Sciences. After assessing the common characteristics of all tea infusions, the panelists rated them based on two aroma attributes (floral and fruity) and two taste attributes (mellow and brisk, sweet aftertaste). A 10-point scale was used for scoring each of the above characteristic, in which 8–10 was represented “extremely strong”, 6–8 “strong”, 4–6 “neutral”, 2–4 “weak” and 0–2 “extremely weak”.

### 2.5 Determination of chromatic parameters (color) of tea infusions

The chromatic parameters of each tea infusion were measured using a spectrophotometer (CM-3500d, Konica Minolta (China) Investment Ltd., Shanghai, China) in a cuboid transparent rectangular cell (size: 50 × 38 mm; optical path: 10 mm), following the methodology described previously (Mao et al., 2021). The color of tea infusion was described using the CIE L*a*b* system. In this system, L* represents the lightness ranging from –100 (dark) to 100 (white), a* indicates the red (+)/green (–) color balance, and b* represents the yellow (+)/blue (–) color balance.

### 2.6 Analysis of non-volatile compounds in tea infusions

#### 2.6.1 Determination of total soluble solids and catechins

After calibrating the refractometer (RX-007α; Atago, Japan) with water, the total soluble solids of the tea infusion were measured.

To determine the concentrations of catechins, gallic acid, and caffeine in the tea infusions, high-performance liquid chromatography with UV detection (HPLC/UV; Shimadzu, Tokyo, Japan) was utilized (Cao et al., 2021b). Prior to injection, the tea infusions were filtered through a 0.45 µm Millipore filter. The chromatographic conditions were set as follows: a Diamonsil C_18_ column (4.6 mm × 250 mm, 5 µm; Dikma Technologies, Lake Forest, CA) was employed, and the column temperature was maintained at 40℃. The detection wavelength was set at 280 nm, and the flow rate was set at 1 mL/min. Mobile phase A consisted of 2 % acetic acid in water, while B was 100 % acetonitrile. The elution gradient started at 6.5 % B at 0 min, gradually increasing to 15 % B at 16 min, remaining at 15 % B until 25 min, and then returning to 6.5 % B at 30 min.

#### 2.6.2 Nontargeted analysis of non-volatile compounds based on UHPLC-Q exactive-MS

UHPLC-Q Exactive-MS (Thermo Fisher Scientific, Rockford, IL) was employed to analyze non-volatile compounds in tea infusions. Prior to injection, the samples underwent filtration using a 0.45 µm Millipore filter. The parameters were configured as follows: electrospray ionization (ESI) in negative ion mode with full scan, utilizing a spray voltage of 3.1 kV. The normalised collision energy was set at 30 %, and the mass scan spanned from a mass/charge (*m*/*z*) ratio of 66.7 to 1,000. The full scan MS and ddMS^2^ resolutions were 70,000 and 35,000, respectively. The capillary and auxiliary gas heater temperatures were 320 °C and 300 °C, respectively, while the sheath and auxiliary gas flow rates were 45 and 10 (in arbitrary units), respectively (Fu et al., 2020).

Separation was carried out on an ACQUITY UPLC HSS T3 column (1.8 μm, 2.1 mm × 100 mm, Waters, Milford, MA, USA) maintained at 40 ℃. The injection volume was 5 μL at a flow rate of 0.3 mL/min, with an injection temperature of 10 ℃. The mobile phases consisted of 0.1 % formic acid in water (A) and 100 % acetonitrile (B). The analysis duration was 12 min, and the mobile phase composition progressed as follows: 5 % B (0–1.0 min), 10 % B (2 min), 35 % B (6 min), 100 % B (8.5–9.5 min), and 5 % B (10.0–12.0 min).

Each sample was analyzed in triplicate. The quality control (QC) sample, used for the stability and repeatability monitoring of the analysis process, was obtained by mixing all tea infusions of the same volume. After data acquisition, Compound Discoverer software was used for initial processing to obtain all the ion fragment information through peak picking and alignment. The obtained data was used for further partial least squares discriminant analysis (PLS-DA) and heat map analysis to screen out the key differential metabolites. The identification of these key differential metabolites was through Human Metabolome Database (https://www.hmdb.ca/), and the relative quantification were conducted based on their peak areas.

### 2.7 Analysis of volatile compounds in tea infusions

#### 2.7.1 Aroma extraction by headspace solid-phase microextraction (HS-SPME)

A 0.5 g tea sample was sealed in a 20 mL glass vial, along with 10 μL of ethyl caprate (internal standard, 10 mg/L), and 5 mL of boiling deionized water was subsequently added. After allowing the mixture to equilibrate for 5 min, the vial was transferred to a 60 ℃ water bath, where the headspace volatiles were absorbed for 60 min using a divinylbenzene/carboxen/poldimethylsiloxane [50/30 μm DVB/CAR/PDMS, Stable flex (2 cm)] coating fiber (Supelco, Inc., Bellefonte, PA, USA). The volatiles were then desorbed in the gas chromatography-mass spectrometry (GC × MS) injector at 250 ℃ for 5 min (Wang et al., 2022).

#### 2.7.2 GC/MS analysis of volatile compounds

Volatile analysis was conducted using an Agilent 6890 gas chromatograph interfaced with an Agilent HP 5975 MSD ion trap mass spectrometer (Wilmington, DE). The system was equipped with a DB-5MS capillary column (30 m × 250 μm × 0.25 μm). The gas chromatography conditions were set as follows: the inlet temperature was maintained at 250 ℃, and high purity helium (99.999 %) was used as the carrier gas at a flow rate of 1.0 mL/min with a split ratio of 15:1. The temperature program was as follows: the initial temperature was set at 40 ℃ and held for 2 min, followed by an increase to 85 ℃ at a rate of 2 ℃/min, and then held at 85 ℃ for an additional 2 min. Subsequently, the temperature was increased to 180 ℃ at a rate of 2.5 ℃/min and held for 2 min. Finally, the temperature was further increased to 230 ℃ at a rate of 10 ℃/min and held for another 2 min. Mass spectrometry (MS) analysis was performed using electron ionization (EI) mode at 70 eV, with a mass scan range of 40–400 *m*/*z*. The ion source temperature was maintained at 230 ℃ (Fu et al., 2020).

#### 2.7.3 Identification and determination of volatile compounds

Volatile compounds were identified by comparing their mass spectra with those in the National Institute of Standards and Technology Mass Spectral Library (NIST14). Additionally, the retention index method was employed for further confirmation. Linear retention indices were determined by injecting *n*-alkanes (C_7_–C_40_) (Alasalvar et al., 2012). The relative concentration of each volatile compound was calculated by comparing its total ion current response in the mass spectrum with that of the internal standard (ethyl decanoate). Odor descriptors for various volatile compounds were obtained from the US National Library of Medicine, accessible at https://pubchem.ncbi.nlm.nih.gov.

### 2.8 Statistical analysis

The data are recorded as mean ± standard deviation (SD) from three independent replicates. Statistical analysis was performed using one-way analysis of variance (ANOVA), followed by the Duncan test to compare the means for significant differences (*p* < 0.05). Partial least squares discriminant analysis (PLS-DA) was conducted in Simca-P (Version 14.1, MKS Umetrics AB, Umeå, Sweden). Figures were plotted with Origin 2021 software (OriginLab, Northampton, MA), GraphPad Prism (Version 9.00, GraphPad Software Inc., San Diego, CA) and TBtools.

## 3 Results and discussion

### 3.1 Physicochemical properties of brewing water samples

**Fig. 1:**
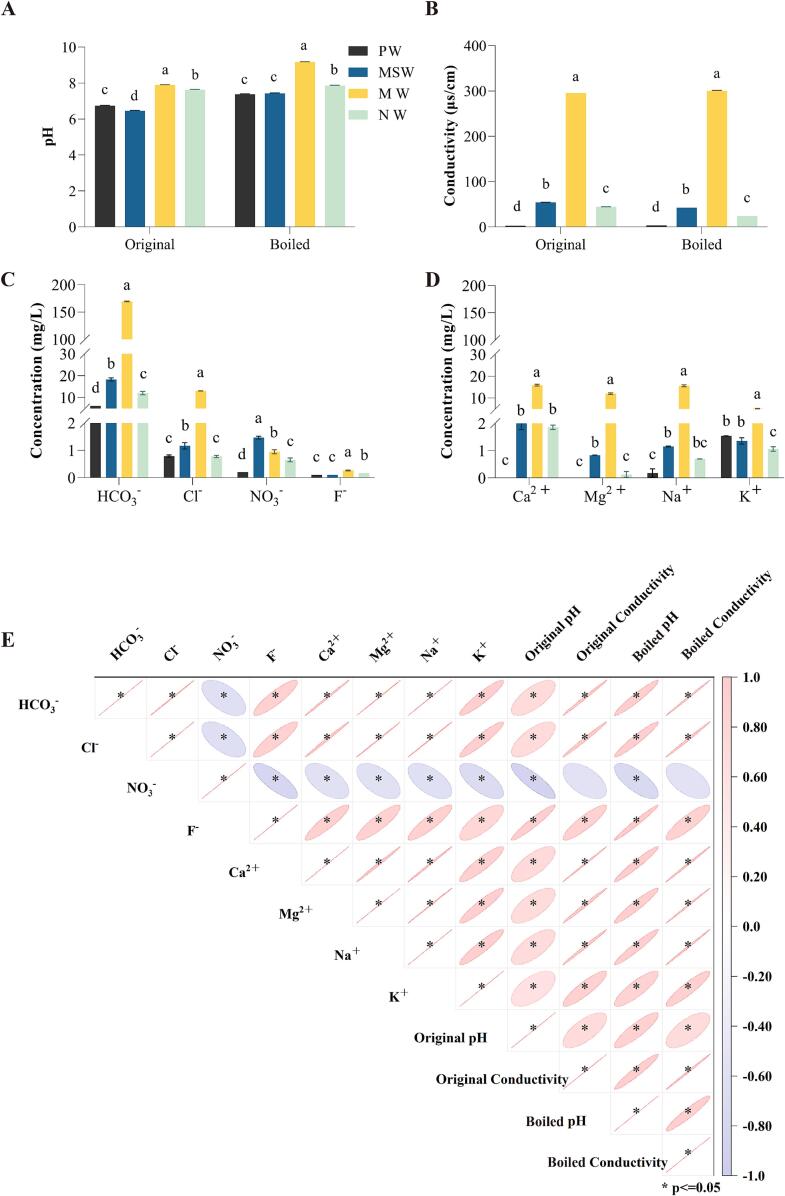
Physicochemical properties of water samples. A & B: the pH & conductivity of waters samples; C & D: the concentrations of anions & metal cations in water samples; E: the correlation of water quality ions with their pH and conductivity. Original & Boiled: water before & after heating. PW: pure water; MSW: mountain spring water; MW: mineral water; NW: natural water. Data are means (±SD) of three replicates. ^a,b,c,d^ Different letters in the same column indicate significant differences between mean values (*p* < 0.05).

As shown in Fig. 1A, significant differences were observed in the pH values of the four water samples (*p* < 0.05). At RT, both MW and NW exhibited pH values above 7.5, while PW and MSW below 7.0. As hot water is commonly used for brewing tea, the pH values of the boiled water samples were measured. It was found that all four water samples showed an up-regulation in pH values after boiling, with an average increase of 0.78, which is consistent with previous studies and mainly due to the lessening of CO_2_ dissolved in water (Cao et al., 2022; Mossion, Potin-Gautier, Delerue, Le Hécho, & Behra, 2008). Regardless of boiling, the pH values of MW remained the highest among all water samples. The conductivity of the four water samples also showed significant differences (*p* < 0.05). In particular, MW had a higher conductivity (300 μs/cm) than the other water samples (*p* < 0.05). Conversely, PW had the lowest conductivity among the samples (Fig. 1B). Overall, PW had lower pH and conductivity, while MW had higher pH and conductivity.

Significant differences were observed in the concentrations of various ions among the four water samples as well, among which MW ranked the first in all the ions content only expect for NO_3_^–^ (Fig. 1C & D). It aligned with the correlation analysis which appeared that all the ions exhibited a positive correlation with the pH and conductivity of the water samples, except for NO_3_^–^ (Fig. 1E). In a sense, water’s acidity and conductivity are dependent on anions and cations within it.

### 3.2 Effect of brewing water on the pH and conductivity of LST and SST infusions

**Fig. 2:**
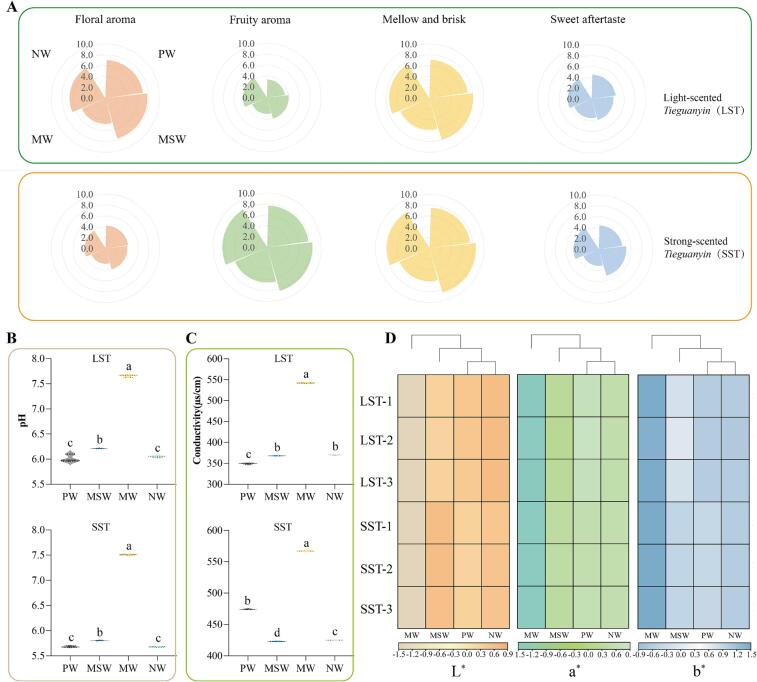
The sensory attributes of tea infusions brewed with four different water samples. A: The flavor evaluation of LST and SST brewed with four different water samples; B: The pH of LST and SST tea infusions; C: The conductivity of LST and SST tea infusions; D: The color of tea infusions of LST and SST brewed with four different water samples. LST: light-scented *Tieguanyin*; SST: strong-scented *Tieguanyin*; PW: pure water; MSW: mountain spring water; MW: mineral water; NW: natural water. Data are means (±SD) of three replicates. ^a,b,c,d^ Different letters in the same column indicate significant differences between mean values (*p* < 0.05).

It is worth noting that the pH values of the tea infusions were lower than the original water samples. In addition, brewing with SST caused a greater decrease of the pH values than with LST (Figs. 1A & 2B). In contrast, the conductivity of the tea infusions increased compared to the original water samples (Fig. 1B), and the conductivity of the SST infusions increased slightly more than the LST infusions (Fig. 2C). Various chemicals releasing from tea leaves were seemed to the main causes, among which some acidic components accounted for the reduction of pH values, such as amino acids, phenols and organic acids, etc., while minerals like Ca^2+^, K^+^ and Mg^2+^, countributed to the rising of conductivity (Xu et al., 2017, Bai et al., 2023). However, regardless of these changes, the pH and conductivity of the tea infusions prepared with MW were still higher than those of the other water samples. Significant positive correlations between tea infusions with water samples could be found for the pH value (r = 0.950, *p* < 0.01) and conductivity (r = 0.998, *p* < 0.01). It appeared that the pH value and conductivity of the tea infusion were mainly depended on its’ brewing water, and the extracts from tea leaves also impacted on that, in some degrees.

### 3.3 Effect of brewing water on the sensory attributes of LST and SST infusions

#### 3.3.1 Effect of brewing water on the sensory quality

Differences between the aroma of LST infusions and SST infusions were observed. Typically, the LST displayed a distinct floral aroma, while the SST exhibited a fruity aroma (Fig. 2A). The distinction might be attributed to the degree of tea leaf roasting (Guo et al., 2021, Cao et al., 2021a).

In addition, the overall taste profiles of the tea infusions prepared with different water samples varied significantly. The LST infusion prepared with MSW received the highest overall acceptability score. Similarly, the SST brewed with MSW and NW displayed similar sensory qualities and achieved high scores. In terms of taste attribute scores, the LST and SST tea infusions brewed with MSW were more mellow and brisk with a sweet aftertaste, while those brewed with MW received the lowest score. In other words, MW had the lowest overall organoleptic quality and was characterized by a flat taste and turbid aroma.

It is reported that the mineral ion influences the sensory quality by flavor interactions (Cabrera et al., 2021). For example, Ca^2+^ could interact with tea polyphenols, caffeine, theanine, and other flavor substances in tea infusion. A high concentration of Ca^2+^ reduces the bitterness, freshness, and sweetness of instant green tea infusion while enhances the astringency (Yin et al., 2014).

#### 3.3.2 Effect of brewing waters on the color

Due to the different fermentation degree, the appearance color of LST and SST infusions varied greatly. LST infusions looked much lighter and greener than SST infusions, but not so yellow as SST. Accordingly, LST infusions were with higher L* values, while lower a* and b* values, comparing with SST.

In addition, brewing water also caused marked differences in the color of the tea infusion (Fig. 2D & Table S1). The tea infusions prepared with MW, the water with the highest pH and conductivity, possessed the lowest L* and a*values, but the highest b*, both for LST and SST. The L* values of the tea infusions showed a significant negative correlation with the pH (r = −0.911, *p* < 0.01) and conductivity (r = −0.978, *p* < 0.01) of the boiled water. It indicated that the higher the pH and of conductivity brewing water was, the darker the appearance color of tea infusion obtained would be. The result was in good agreement with previous studies, because the high pH value and mineralization of water caused the oxidation of catechin, resulting in darker color of tea infusion (Fan et al., 2016, Cao et al., 2021b, Deng et al., 2023). Mineralization in this context refers to the sum of carbonates, bicarbonates, chlorides, sulphates, nitrates and sodium salts of metals such as calcium, magnesium, aluminium and manganese in the water.

### 3.4 Effects of brewing water on the nonvolatile components of LST and SST infusions

#### 3.4.1 Effect of brewing water on the total soluble solids content and catechin concentrations

**Fig. 3:**
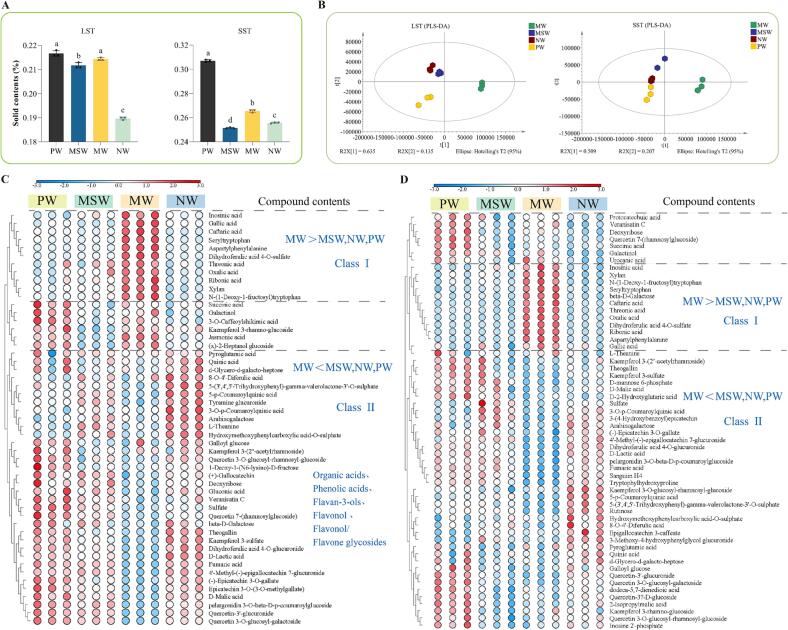
The solid content in tea infusions brewed with four different water samples and the analysis results of PLS-DA of the non-targeted components. A: The solid content of LST and SST brewed with four different water samples respectively; B: The score scatter plots of PLS-DA of LST and SST; C & D: Heat maps of non-targeted components with VIP ≥ 1 in LST and SST. LST: light-scented *Tieguanyin*; SST: strong-scented *Tieguanyin*; PW: pure water; MSW: mountain spring water; MW: mineral water; NW: natural water. Data are means (±SD) of three replicates. ^a,b,c,d^ Different letters in the same column indicate significant differences between mean values (*p* < 0.05).

The substance leached into tea infusion is influenced not only by the inherent quality of the tea leaves, but also by various brewing factors, with the type of brewing water plays a crucial role. In this study, significant differences in the total soluble solids content were observed among tea infusions brewed with different water samples (Fig. 3A). The total soluble solids contents of LST and SST infusions varied with different water types, following the order PW, MW > MSW > NW.

Catechins, a crucial class of phenolic compounds in tea influencing the flavor, antioxidant, and other bioactivities, can be categorized into *epi*- catechins (including EC, EGC, ECG and EGCG) and non-*epi*-catechins (including C, GC, CG, and GCG). The brewing water quality significantly influenced the concentrations of catechins in tea infusions (Table. 1). LST and SST infusions brewed with MW both contained the lowest *epi*-catechins compared to the other three water types. The highest *epi*-catechin concentration in LST brewed with MSW (276.48 mg/L) was highest, followed by PW (263.46 mg/L), NW (260.70 mg/L), and MW (82.25 mg/L). In particular, the EGCG content was the most significant difference, with similar results were observed for SST. Correlation analysis revealed a significant negative association between the pH and conductivity of water samples and the concentrations of *epi*-catechins in tea infusions (Tables S2 & S3). For example, in SST, EGCG showed a significant negative correlation with boiled pH and conductivity (−0.951 and −0.997, respectively, *p* < 0.01), with similar results were observed in LST. This suggests that the high pH and conductivity of MW adversely affected the leaching of catechins.

**Table 1:** Catechins content of two *Tieguanyin* oolong teas brewed with different water samples.

Content (mg/L)	Light-scented *Tieguanyin* (LST)				Strong-scented *Tieguanyin* (SST)			
	PW	MSW	MW	NW	PW	MSW	MW	NW
GC	22.590 ± 0.391 ^a^	5.517 ± 0.110^b^	5.670 ± 0.134^b^	17.136 ± 5.216 ^a^	4.086 ± 0.042 ^d^	4.992 ± 0.139^c^	6.967 ± 0.361^b^	83.219 ± 0.100 ^a^
EGC	67.856 ± 0.126 ^a^	59.770 ± 0.028^c^	6.254 ± 0.176 ^d^	63.663 ± 1.389^b^	67.338 ± 0.197 ^a^	56.360 ± 0.148^c^	4.072 ± 0.285 ^d^	64.149 ± 0.081^b^
C	1.021 ± 0.026^b^	0.475 ± 0.023^c^	4.630 ± 0.00 ^a^	1.085 ± 0.192^b^	0.639 ± 0.030^c^	0.511 ± 0.008 ^d^	2.112 ± 0.020 ^a^	1.486 ± 0.098^b^
EGCG	131.124 ± 3.568^b^	144.268 ± 5.021 ^a^	27.487 ± 1.760^c^	133.404 ± 4.017^b^	178.181 ± 6.354 ^a^	146.288 ± 2.200^c^	22.606 ± 1.421 ^d^	162.880 ± 4.561^b^
EC	44.817 ± 0.389^b^	49.536 ± 2.649 ^a^	32.222 ± 0.062^c^	43.488 ± 0.114^b^	48.768 ± 4.143 ^a^	41.577 ± 3.507^b^	28.394 ± 0.001^c^	50.579 ± 0.154 ^a^
GCG	0.600 ± 0.037 ^d^	2.420 ± 0.096^b^	7.037 ± 0.483 ^a^	1.298 ± 0.145^c^	0.631 ± 0.041^c^	1.043 ± 0.045^b^	6.357 ± 0.452 ^a^	1.312 ± 0.077^b^
ECG	19.658 ± 0.686^b^	22.908 ± 0.785 ^a^	16.281 ± 0.738^c^	20.144 ± 0.872^b^	23.711 ± 0.688 ^a^	20.671 ± 0.732^c^	15.117 ± 0.360 ^d^	22.193 ± 0.801^b^
CG	0.713 ± 0.011^b^	0.752 ± 0.011 ^a^	0.302 ± 0.033 ^d^	0.590 ± 0.000^c^	0.969 ± 0.017 ^a^	0.942 ± 0.001^b^	0.169 ± 0.009^c^	0.975 ± 0.005 ^a^
*Epi*-catechins	263.46 ± 4.77 ^a^	276.48 ± 3.13 ^a^	82.25 ± 2.74^c^	260.70 ± 3.39^b^	318.00 ± 3.10 ^a^	264.90 ± 6.29^b^	70.19 ± 2.07^c^	299.80 ± 5.60 ^a^
Non-*epi*-catechins	24.92 ± 0.39 ^a^	9.16 ± 0.02^b^	17.64 ± 0.38 ^ab^	20.11 ± 5.17 ^a^	6.33 ± 0.13^c^	7.49 ± 0.19^c^	15.60 ± 0.84^b^	86.99 ± 0.07 ^a^
Total catechins	288.38 ± 5.16 ^a^	285.64 ± 3.15 ^a^	99.88 ± 3.12^b^	277.12 ± 13.77 ^a^	324.32 ± 3.23^b^	272.38 ± 6.48^c^	85.79 ± 2.91 ^d^	386.79 ± 5.67 ^a^

LST: light-scented *Tieguanyin*; SST: strong-scented *Tieguanyin*; PW: pure water; MSW: mountain spring water; MW: mineral water; NW: natural water. Data are means (±SD) of three replicates. ^a,b,c,d^ Different letters in the same row indicate the significant difference among the four water samples (*p* < 0.05).

Galloylated catechins, especially EGCG and ECG, play a significant role in imparting bitterness and astringency to tea infusion. It was found that EGCG and ECG concentrations containing in tea infusion were negatively correlated (*p* < 0.01) with the concentrations of cations and anions in the water samples, except for ECG with NO_3_^–^ (Tables S4 & S5). It can be inferred that elevated mineral ions in brewing water resulted in reduced levels of galloylated catechins in the infusion, leading to a diminished bitter and astringent taste of tea infusion. This finding partially explains why tea infusions brewed with MW exhibited a weaker taste. These results underscore intricate connection between the ion concentration of the brewing water and the flavor profile of tea infusions.

#### 3.4.2 Effect of brewing waters on the non-targeted compounds

Non-targeted metabolomic analysis of *Tieguanyin* tea infusions brewed with different water samples was performed using UHPLC-Q Exactive mass spectroscopy, and the ionic signatures of 1290 unknowns were obtained. After screening the blank samples and quality control to eliminate blank background and spurious signals, PLS-DA was applied to discriminate the compositional differences among the *Tieguanyin* tea infusions brewed with the four water samples. The model predictor Q^2^ (cum) for LST infusions was 0.974, and the fit indicators for the independent variable R^2^X (cum) and the dependent variable R^2^Y (cum) were 0.93 and 0.996, respectively. The model predictor Q2 (cum) for SST infusions was 0.939, and the fit indicators for the independent variable R^2^X (cum) and the dependent variable R^2^Y (cum) were 0.904 and 0.993, respectively. Meanwhile, the predictive ability of permutation test without overfitting phenomenon (validation of the model was repeated for 200 times of calculations) (Fig. S1A & B) further confirmed the reliability of the PLS-DA model.

As shown in the PLS-DA scoring plot (Fig. 3B), the tea infusions of LST brewed with the four water samples were roughly divided into three blocks, with MSW and NW as a whole were clustered in the upper left, PW alone in the lower left, and MW alone in the right. The tea infusions of SST were mainly divided into two parts, in which PW, MSW and NW as a whole clustered on the left, and MW alone clustered on the right. This suggests that for both LST and SST, the chemical compositions of tea infusions prepared by MW were different from those of infusions prepared with the other three water samples. The differential compounds might be the key substances leading to the differences in color and flavor.

The key differential compounds were screened out with Variable Importance in Projection (VIP) greater than 1 in SIMCA P, shown as Fig. 3C & D. The clustering of compounds in LST and SST tea infusions were roughly divided into two major parts, in which the class I were mostly dominated by acids class, including organic acids (oxalic acid) and phenolic acid (gallic acid, caffeic acid), with an overall distribution trend of MW > MSW, NW, PW. A previous study reported that sulfates and chlorides, bicarbonates of calcium and magnesium reacted with the tea components to produce a turbid infusion with a flat taste and dull color (Pangborn et al., 1971). In addition, Ca^2+^ formed precipitates with oxalic acid, and tartaric acid when the mass concentration of Ca^2+^ in drinking water was greater than 40 mg/L, making the tea infusion turbid (Xu et al., 2013). Tea infusions brewed by MW were with high contents of mineral ions, oxalic acid. The organic acids and metal ions in the tea infusions could form precipitation, which was conjectured to lower the lightness of tea infusions brewed by MW. Khan et al. (2019) found that metal ions formed complexes with gallic acid, such as gallate, and affected the flavor of the tea infusion. It suggested that the relatively high levels of gallic acid in the tea infusions brewed by MW could also influence the taste.

On the contrary, the distribution trend of the class II of compounds clustering was MW < MSW, NW, PW. Compounds in the second clustering were mainly flavan-3-ols, flavonol and flavone /flavonol glycosides, organic acids, phenolic acids and their derivatives. These compounds were also identified as the metabolites responsible for the differences between *Tieguanyin* teas produced in spring and autumn (Zhou et al., 2019). A previous study claimed that the main contributors to the taste of *Tieguanyin* oolong tea were catechins, theaflavins, flavonol glycosides and caffeine (Liu et al., 2018). It was consistent with our results. Among them, flavan-3-ols were one class of the key components in tea. They not only displayed a variety of biological activities such as anti-inflammatory (Yang et al., 2022), but were also involved in the bitterness and astringency of tea infusion (Yang et al., 2018). Flavonol and flavone /flavonol glycosides, such as quercetin and its derivatives, were the main flavor compounds influencing the astringency of tea infusions (Zhao et al., 2017). In terms of their distribution, PW, MSW and NW were all more beneficial than MW for the release of flavor compounds from *Tieguanyin* tea, which manifested as better flavor of tea infusions prepared by the former three types of water.

However, different tea extracts have a greater impact on the overall harmony of the tea infusion due to the different content of flavoring substances and the threshold of flavor presentation. For example, compared to catechins, flavon-3-ol glycosides developed a silky and mouth-drying sensation at a very low threshold concentration of 0.001–19.8 μmol/L (Shen et al., 2022). Although present at low levels, flavone/flavonol glycosides have been identified as key compounds that produce a velvety astringency in tea infusions due to their extremely low flavor presentation threshold (Chen et al., 2020) and increase bitter taste by interacting with caffeine (Zhang et al., 2020). In contrast, LST (Fig. 3C) and SST (Fig. 3D) tea infusions brewed with PW contained relatively more flavone/flavonol glycosides. This is consistent with the sensory results that the MSW and NW flavor attributes were relatively higher and the sensory experience was better.

Organic and phenolic acids were the major acids in tea, and their associated metabolites influenced the flavor quality of tea. For example, organic acids in tea were closely related to the acidity and fruitiness flavor of green tea, and were crucial intermediate metabolites in the tricarboxylic acid cycle and shikimic acid pathway (Yu et al., 2022). Similarly, the involvement of organic acids was essential in the formation of the flavor characteristics of *Tieguanyin* tea infusions. It was found that isocitric acid and malic acid were more prominent in *Tieguanyin* tea in spring, which contributed to umami taste of *Tieguanyin* tea infusions (Istiqamah et al., 2019). Quinic acid and myricetin 3-*O*-galactosylrutinoside also benefited the freshness of *Tieguanyin* (Zhou et al., 2019). In addition, succinic acid was identified as an umami-enhancing compound (Kaneko et al., 2006). The aroma contributors of autumn *Tieguanyin* were found to be associated with 4-caffeoylquinic acid, 3-p-coumaroylquinic acid, 5-p-coumaroylquinic acid and sanguiin H4, which suggested a correlation between the quality traits of “floral and fruity aroma” of *Tieguanyin* and phenolic acids (Pękal et al., 2011).

In summary, mineral ions in the brewing water influenced the flavor and color of tea infusions through complexation with tea components. The *Tieguanyin* tea infusions brewed with MW exhibited a flat taste and turbid aroma, which was mainly due to the low leaching of flavor components to the tea infusions during the brewing process, such as flavan-3-ols, flavonol and flavone/flavonol glycosides, organic acids, phenolic acids and other substances. Compared to LST infusions, the flavor components in SST infusions were less relatively, which could be attributed to the different roasting degree of SST and LST. A previous study indicated that the intensity of bitterness and astringency of tea gradually decreased with increasing roasting temperature (Jiang et al., 2022). This was consistent with the sensory evaluation results, in which LST was slightly more astringent and less mellow than SST.

### 3.5 Effects of brewing water on the volatile components of LST and SST infusions

#### 3.5.1 Identification and overview of volatile compounds

**Fig. 4:**
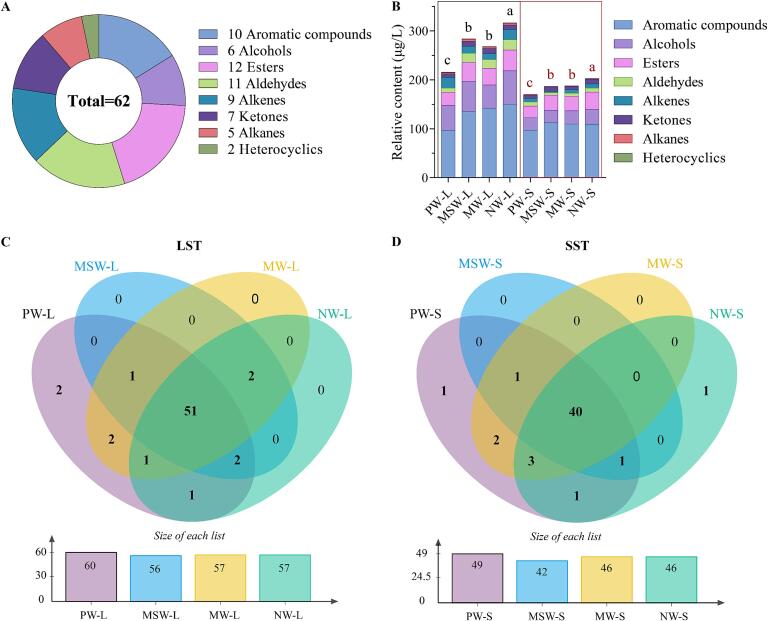
Overview of volatile components in tea infusions brewed with four different water samples. A: Quantitative distribution of chemical classes of volatile compounds; B: Relative abundance of different types of volatile compounds; C & D: Venn plot of LST and SST. LST: light-scented *Tieguanyin*; SST: strong-scented *Tieguanyin*; PW: pure water; MSW: mountain spring water; MW: mineral water; NW: natural water. The numbers in the Venn plot represent the number of identified metabolites. Data are means (±SD) of three replicates. ^a,b,c,d^ Different letters in the same column indicate significant differences between mean values (*p* < 0.05).

In this study, volatile compounds were analyzed by SPME/GC–MS in tea samples brewed with four different types of water. A total of 62 volatile compounds were identified in the tea infusions, including 12 esters, 11 aldehydes, 10 aromatics, 9 alkenes, 7 ketones, 6 alcohols, and 2 heterocyclics (Fig. 4A). Differences in aroma components were observed between LST and SST when using different water samples. Specifically, 51 compounds were identified in LST across all four water samples, while 40 compounds were identified consistently in SST (Fig. 4C & D). The small numbers of volatile compounds found in the SST were attributed to the gradual dissipation of volatile compounds resulting from higher temperatures and longer roasting conditions (Cao et al., 2021a).

The relative content of volatile compounds calculated from the total ion chromatograms varied in the concentration and proportion of each chemical class in different samples (Fig. 4B & Table S6). As shown in Fig. 4B, the overall relative contents of total volatile compounds were higher in LST brewed with the four waters than in SST. Previous studies showed that a prolonged high-temperature roasting process leads to the dissipation of floral volatiles (e.g., *β*-ionone, jasmine, and nerolidol) and green volatiles (e.g., *cis*-3-hexenyl isovalerate, heptanal) components in SST (Gao et al., 2022). In addition, the type of brewing water had a greater effect on the relative content of total volatile compounds in LST than that in SST. The LST brewed with NW had the highest level of total volatile compounds (316.71 μg/L), which was significantly (*p* < 0.05) higher than LST brewed with the other three waters. It was followed by MSW and MW, which reached 283.82 μg/L and 268.15 μg/L, respectively, significantly higher than 215.67 μg/L brewed with PW (*p* < 0.05). A similar trend was observed in SST, where the LST brewed with NW had the highest content of total volatile compounds (202.78 μg/L), which was significantly higher than that brewed with PW (170.24 μg/L) (*p* < 0.05). A recent study showed that Ca^2+^ in the brewing water had a significant effect on the release of aroma compounds in tea infusion. When the Ca^2+^ concentration was less than 30 mg/L, the aroma content increases with Ca^2+^ concentration; while the aroma content decreases, when more than 30 mg/L (Bai et al., 2023). In the present study, PW and MW had lower aroma contents, supporting the hypothesis that the brewing water which contained too low or too high Ca^2+^ concentration was not conducive to the dissemination of tea aroma.

Indole (73.44–114.28 μg/L), nerolidol (23.84–65.37 μg/L), benzeneacetaldehyde (0.00–16.45 μg/L), and (*Z*)-*β*-farnesene (0.00–11.69 μg/L) were identified as the major volatile constituents in LST and SST (Table S6). These compounds were reported to be the characteristic components of oolong tea with floral and sweet aroma (Zeng et al., 2022b). The content of the four volatiles in LST was higher than that in SST, which well explained why LST exhibited more noticeable floral sensory characteristics.

#### 3.5.2 Analysis of the effect of different types of brewing water on volatiles

**Fig. 5:**
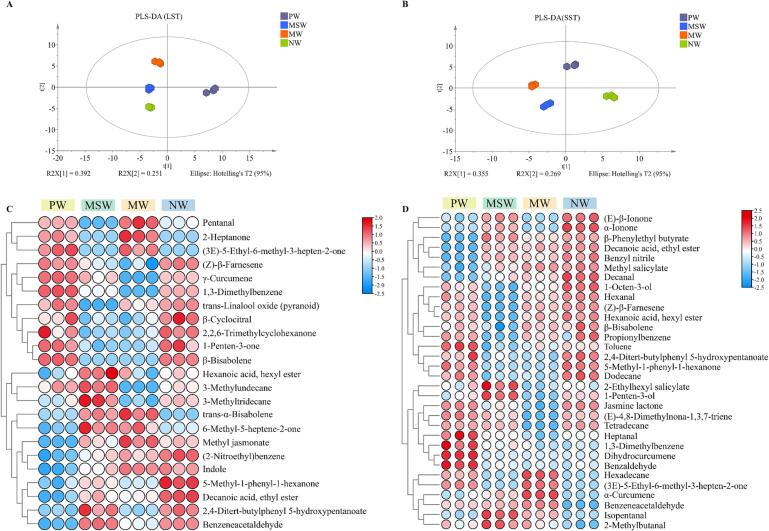
The analysis results of PLS-DA of the volatile compounds in tea brewed with four different water samples. A: Score plot of PLS-DA of LST; B: Score plot of PLS-DA of SST; C: Heat maps of volatiles with VIP ≥ 1 in LST; D: Heat maps of volatiles with VIP ≥ 1 in SST. LST: light-scented *Tieguanyin*; SST: strong-scented *Tieguanyin*; PW: pure water; MSW: mountain spring water; MW: mineral water; NW: natural water.

Although multiple volatile compounds were identified in oolong tea samples brewed using different types of water, not all of them play a significant role in the discriminant analysis. To fully understand the effects of the four different types of water on the volatile compounds in the brewed tea samples, the PLS-DA analysis was performed based on the relative contents of 62 volatile compounds. In LST, along the direction of principal component 1 (R^2^X [1] = 0.392) and the direction of principal component 2 (R^2^X [2] = 0.251), there was a clear separation between these clusters (Fig. 5A). The PLS-DA model was validated through 200 permutation tests, confirming that the model was not overfitting (Fig. S1C & D). The model had high predictive power (Q^2^ (cum) = 0.942). The clear separation and high reproducibility between the different sample groups indicated significant differences in the volatile components. Generally, compounds with VIP ≥ 1 after PLS-DA analysis were recognized as important contributors to the variations in tea aroma. Using the PLS-DA model, 23 key volatile compounds with VIP ≥ 1 across all samples were screened (Fig. 5C). In SST, along the direction of principal component 1 (R^2^X [1] = 0.355), and along the direction of principal component 2 (R^2^X [2] = 0.269), the four samples were clearly separated from each other (Fig. 5B). The model was not overfitted and had high predictive power (Q^2^ (cum) = 0.976) and good predictive power. By employing the PLS-DA model, a total of 32 significant volatile compounds from all samples, based on their VIP ≥ 1, were successfully screened (Fig. 5D). It suggested that the choice of brewing water was important for the aroma profile of oolong tea.

For LST, the four different types of brewing water significantly influenced the volatile components. For instance, benzeneacetaldehyde, known for its roses-like floral aroma and reported as the main odorant of Keemun black tea (Kang et al., 2019). Its content in LST brew with MSW and NW (16.45 ug/L and 15.23 ug/L, respectively) was significantly higher (*p* < 0.05) than that in PW and MW (4.83ug/L and 9.16 μg/L, respectively). Nerolidol, recognized for its floral and citrus aroma, was identified as one of the key aroma components of *Tieguanyin* oolong tea (Cao et al., 2021a), also showed significantly higher content in LST brewed with MSW and NW (58.99 μg/L and 65.37 μg/L, respectively) than that in PW and MW (48.87 μg/L and 45.08 μg/L, respectively) (*p* < 0.05). In addition, *α*-ionone and (*E*)-*β*-ionone, known for their violet floral aroma with woody and fruity odors as well, with very low thresholds (Yang et al., 2023b). Their contents were the highest in both LST and SST using NW for brewing, and lower in tea infusion using MW for brewing. Other volatile compounds, such as benzyl nitrile, decanoic acid, ethyl ester, and linalool, followed similar patterns.

Compared to the other three types of water, MW had higher levels of pH and minerals, including Ca^2+^, Mg^2+^, and Na^+^. Li et al. (2023) found that water with high pH and ionic concentration inhibited the release of floral compounds (such as *β*-ionone and, linalool) and fresh compounds (such as dimethyl sulfide and, hexanal) during tea brewing. The presence of Fe^2+^ in green tea infusion resulted in the loss of green and grassy volatiles and the formation of new aroma compounds (Gao et al., 2021). In addition, it was also found that both acidic and alkaline brewing water reduced the aroma content of tea. When the pH value of the brewing water was increased from 4.5 to 6.5, the aroma content of the tea increased; but further increased to above 7.5, the aroma content decreased (Bai et al., 2023). Overall, our study found that brewing *Tieguanyin* oolong tea using NW and MSW water were favorable for improving its aroma quality, while the opposite effects were observed for MW and PW. This might attributed to the moderate levels of minerals (e.g., Ca^2+^, Mg^2+^, and Na^+^) in MSW and NW. However, it is well known that tea aroma is affected by a multitude of factors, and more systematic studies are needed to elaborate on the effect of different types of water on volatile components of tea.

## 4 Conclusions

Brewing *Tieguanyin* oolong tea with four water samples of varying physicochemical properties resulted in significant differences in the flavor profile of the tea infusion and the concentrations of the tea components. Infusions prepared with MW (with high pH and mineralization) exhibited a flat taste and turbid aroma. The leaching of key flavor components, including flavanols, flavonols and flavonol glycosides, organic acids, phenolic acids and other substances, was relatively low in the tea infusion brewed by MW, hindering the expression of *Tieguanyin* tea’s distinctive flavors. Excessive or insufficient ion content affected the relative content of volatile compounds, negatively affecting the purity and richness, and diminishing sensory quality. Therefore, it is recommended to utilize water samples with moderate pH and ion concentration, such as NW and MSW, for brewing *Tieguanyin* oolong tea to ensure an optimal sensory experience. These findings provide a valuable foundation for tea beverage manufacturers to improve flavor attributes and minimize flavor loss, and guide consumers in selecting optimal brewing water.

## CRediT authorship contribution statement

**Yuan-Yuan Ma:** Data curation, Investigation, Writing – original draft, Writing – review & editing. **Jie-Qiong Wang:** Data curation, Formal analysis, Methodology, Software. **Ying Gao:** Writing – review & editing. **Qing-Qing Cao:** Conceptualization, Methodology, Validation, Writing – review & editing. **Fang Wang:** Investigation. **Jian-Xin Chen:** Investigation. **Zhi-Hui Feng:** Investigation. **Jun-Feng Yin:** Conceptualization, Visualization, Writing – review & editing. **Yong-Quan Xu:** Conceptualization, Funding acquisition, Investigation, Supervision, Writing – review & editing.

## Declaration of competing interest

The authors declare that they have no known competing financial interests or personal relationships that could have appeared to influence the work reported in this paper.

## Data Availability

The data that has been used is confidential.
