# Supplementary material for: Effect of the type of brewing water on the sensory and physicochemical properties of light-scented and strong-scented Tieguanyin oolong teas
Source: Food Chem X. 2023 Dec 23;21:101099. doi: 10.1016/j.fochx.2023.101099 (PMC10792187; doi:10.1016/j.fochx.2023.101099)
Supplement: Supplementary data 1 [file mmc1.docx]

**Effect of the type of brewing water on the sensory and physicochemical properties of** **light-scented and strong-scented *Tieguanyin* oolong teas**

Yuan-Yuan Ma ^1, 2, a^，Jie-Qiong Wang ^1, 2, a^，Ying Gao ^1^， Qing-Qing Cao ^1*^,

Fang Wang ^1^, Jian-Xin Chen ^1^，Zhi-Hui Feng ^1^, Jun-Feng Yin ^1^, Yong-Quan Xu ^1**^

^1^ *Tea Research Institute, Chinese Academy of Agricultural Sciences, Key Laboratory of Biology, Genetics and Breeding of Special Economic Animals and Plants, Ministry of Agriculture and Rural Affairs, 9 South Meiling Road, Hangzhou 310008, China*

^2^ *Graduate School of Chinese Academy of Agricultural Sciences, Beijing 100081, China*

^a^These authors contributed equally.

**Corresponding Author**

^**^ Yong-Quan Xu, Tel: +86-571-86017633. Fax: +86 571 86650056. Email: [yqx33@126.com](mailto:yqx33@126.com).

^*^ Qing-Qing Cao, Tel: +86-571-86650594. Fax: +86 571 86650056. Email: [caoqingqing@tricaas.com](mailto:caoqingqing@tricaas.com).

**Table S1.** The color of two *Tieguanyin* oolong teas brewed with different water samples

|  | Light-scented *Tieguanyin* (LST) | | | | Strong-scented *Tieguanyin* (SST) | | | |
| --- | --- | --- | --- | --- | --- | --- | --- | --- |
|  | \| PW \| \| --- \| | MSW | MW | NW | \| PW \| \| --- \| | MSW | MW | NW |
| L* | 98.967±0.042^a^ | 98.787±0.012^a^ | 96.967±0.341^b^ | 99.153±0.012^a^ | 97.140±0.010^c^ | 97.617±0.023^a^ | 94.510±0.036^d^ | 97.467±0.006^b^ |
| a* | -1.467±0.012^a^ | -2.040±0.010^b^ | -4.203±0.436^c^ | -1.717±0.015^ab^ | -1.650±0.020^a^ | -1.750±0.020^b^ | -2.833±0.006^c^ | -1.660±0.010^a^ |
| b* | 5.560±0.036^b^ | 8.050±0.036^b^ | 17.313±2.333^a^ | 5.323±0.015^b^ | 11.930±0.000^b^ | 11.457±0.031^c^ | 22.637±0.031^a^ | 10.713±0.040d |

LST: light-scented *Tieguanyin*; SST: strong-scented *Tieguanyin*; PW: pure water; MSW: mountain spring water; MW: mineral water; NW: natural water. Data are means (± SD) of three replicates. ^a,b,c,d^ Different letters in the same row indicate significant differences between mean values (*p* < 0.05).

**Table S2.** Correlation of epi-catechins concentrations in LST tea infusions with water pH and conductivity

|  | Boiled pH | Boiled Conductivity | EC | EGC | ECG | EGCG |
| --- | --- | --- | --- | --- | --- | --- |
| Boiled pH | 1 |  |  |  |  |  |
| Boiled Conductivity | .963^**^ | 1 |  |  |  |  |
| EC | -.952^**^ | -.893^**^ | 1 |  |  |  |
| EGC | -.963^**^ | -1.000^**^ | .893^**^ | 1 |  |  |
| ECG | -.842^**^ | -.775^**^ | .936^**^ | .774^**^ | 1 |  |
| EGCG | -.966^**^ | -.976^**^ | .953^**^ | .975^**^ | .892^**^ | 1 |

Note: LST: light-scented *Tieguanyin*; Boiled: water after heating; Correlation ** indicates (*p* < 0.01).

**Table S3.** Correlation of epi-catechins concentrations in SST tea infusions with water pH and conductivity

|  | Boiled pH | Boiled Conductivity | EC | EGC | ECG | EGCG |
| --- | --- | --- | --- | --- | --- | --- |
| Boiled pH | 1 |  |  |  |  |  |
| Boiled Conductivity | .963^**^ | 1 |  |  |  |  |
| EC | -.820^**^ | -.934^**^ | 1 |  |  |  |
| EGC | -.948^**^ | -.999^**^ | .949^**^ | 1 |  |  |
| ECG | -.910^**^ | -.969^**^ | .945^**^ | .976^**^ | 1 |  |
| EGCG | -.951^**^ | -.997^**^ | .943^**^ | .998^**^ | .985^**^ | 1 |

Note: SST: strong-scented *Tieguanyin*; Boiled: water after heating; Correlation ** indicates (*p* < 0.01).

**Table S4.** Correlation of EGCG and ECG concentrations in LST tea infusions with water quality ions

|  | HCO_3_^-^ | Cl^-^ | NO_3_^-^ | F^-^ | Ca^2＋^ | Mg^2＋^ | Na^＋^ | K^＋^ | ECG | EGCG |
| --- | --- | --- | --- | --- | --- | --- | --- | --- | --- | --- |
| HCO_3_^-^ | 1 |  |  |  |  |  |  |  |  |  |
| Cl^-^ | .999^**^ | 1 |  |  |  |  |  |  |  |  |
| NO_3_^-^ | .223 | .190 | 1 |  |  |  |  |  |  |  |
| F^-^ | .898^**^ | .893^**^ | .077 | 1 |  |  |  |  |  |  |
| Ca^2＋^ | .996^**^ | .993^**^ | .264 | .919^**^ | 1 |  |  |  |  |  |
| Mg^2＋^ | .999^**^ | .999^**^ | .222 | .891^**^ | .995^**^ | 1 |  |  |  |  |
| Na^＋^ | .999^**^ | .999^**^ | .213 | .902^**^ | .996^**^ | 1.000^**^ | 1 |  |  |  |
| K^＋^ | .840^**^ | .854^**^ | -.059 | .685^*^ | .809^**^ | .846^**^ | .838^**^ | 1 |  |  |
| ECG | -.804^**^ | -.819^**^ | .362 | -.840^**^ | -.782^**^ | -.802^**^ | -.810^**^ | -.764^**^ | 1 |  |
| EGCG | -.985^**^ | -.990^**^ | -.060 | -.911^**^ | -.975^**^ | -.985^**^ | -.987^**^ | -.860^**^ | .892^**^ | 1 |

Note: LST: light-scented *Tieguanyin*; Correlation ** indicates (*p* < 0.01) and * indicates (*p* < 0.05).

**Table S5.** Correlation of EGCG and ECG concentrations in SST tea infusions with water quality ions

|  | HCO_3_^-^ | Cl^-^ | NO_3_^-^ | F^-^ | Ca^2＋^ | Mg^2＋^ | Na^＋^ | K^＋^ | ECG | EGCG |
| --- | --- | --- | --- | --- | --- | --- | --- | --- | --- | --- |
| HCO_3_^-^ | 1 |  |  |  |  |  |  |  |  |  |
| Cl^-^ | .999^**^ | 1 |  |  |  |  |  |  |  |  |
| NO_3_^-^ | .223 | .190 | 1 |  |  |  |  |  |  |  |
| F^-^ | .898^**^ | .893^**^ | .077 | 1 |  |  |  |  |  |  |
| Ca^2＋^ | .996^**^ | .993^**^ | .264 | .919^**^ | 1 |  |  |  |  |  |
| Mg^2＋^ | .999^**^ | .999^**^ | .222 | .891^**^ | .995^**^ | 1 |  |  |  |  |
| Na^＋^ | .999^**^ | .999^**^ | .213 | .902^**^ | .996^**^ | 1.000^**^ | 1 |  |  |  |
| K^＋^ | .840^**^ | .854^**^ | -.059 | .685^*^ | .809^**^ | .846^**^ | .838^**^ | 1 |  |  |
| ECG | -.956^**^ | -.944^**^ | -.469 | -.846^**^ | -.966^**^ | -.955^**^ | -.954^**^ | -.704^*^ | 1 |  |
| EGCG | -.992^**^ | -.986^**^ | -.337 | -.883^**^ | -.994^**^ | -.991^**^ | -.991^**^ | -.788^**^ | .985^**^ | 1 |

Note: SST: strong-scented *Tieguanyin*; Correlation ** indicates (*p* < 0.01) and * indicates (*p* < 0.05).

**Table S6.** Volatiles compounds content of two *Tieguanyin* oolong teas brewed with different water samples

| **No.** | **Compounds** | **CAS** | **RI**  **(Ref)** | **RI**  **(Cal)** | **Flavor** | **LST Relative content (μg/L)** | | | | **SST Relative content (μg/L)** | | | |
| --- | --- | --- | --- | --- | --- | --- | --- | --- | --- | --- | --- | --- | --- |
|  |  |  |  |  |  | **PW** | **MSW** | **MW** | **NW** | **PW** | **MSW** | **MW** | **NW** |
| Alcohols | | | | | | | | | | | | | |
| 1 | 1-Penten-3-ol | 616-25-1 | 684 | 672 | / | 0.29 ± 0.05 ^a^ | 0.19 ± 0.02 ^a^ | 0.23 ± 0.07 ^a^ | 0.23 ± 0.01 ^a^ | 0.34 ± 0.04 ^b^ | 0.47 ± 0.02 ^a^ | 0.25 ± 0.02 ^c^ | 0.39 ± 0.00 ^b^ |
| 2 | 1-Pentanol | 71-41-0 | 765 | 805 | Balsam, oil, sweet | 0.19 ± 0.04 ^a^ | 0.16 ± 0.02 ^a^ | 0.18 ± 0.01 ^a^ | 0.20 ± 0.01 ^a^ | 0.15 ± 0.01 ^ab^ | 0.15 ± 0.01 ^a^ | 0.13 ± 0.02 ^ab^ | 0.12 ± 0.00 ^b^ |
| 3 | 1-Octen-3-ol | 3391-86-4 | 980 | 991 | / | 0.29 ± 0.05 ^a^ | 0.28 ± 0.02 ^a^ | 0.35 ± 0.02 ^a^ | 0.29 ± 0.01 ^a^ | 0.33 ± 0.01 ^b^ | 0.26 ± 0.01 ^c^ | 0.32 ± 0.00 ^b^ | 0.37 ± 0.01 ^a^ |
| 4 | Linalool | 78-70-6 | 1099 | 1104 | Floral, fresh, woody | 1.94 ± 0.07 ^b^ | 2.01 ± 0.30 ^b^ | 1.78 ± 0.10 ^b^ | 2.57 ± 0.09 ^a^ | 0.85 ± 0.00 ^b^ | 0.97 ± 0.04 ^ab^ | 0.85 ± 0.01 ^b^ | 1.18 ± 0.18 ^a^ |
| 5 | trans-Linalool oxide (pyranoid) | 39028-58-5 | 1173 | 1171 | / | 0.11 ± 0.01 ^a^ | 0.00 ± 0.00 ^c^ | 0.08 ± 0.01 ^b^ | 0.11 ± 0.00 ^a^ | 0.00 ± 0.00 | 0.00 ± 0.00 | 0.00 ± 0.00 | 0.00 ± 0.00 |
| 6 | Nerolidol | 7212-44-4 | 1564 | 1565 | Floral, citrus, waxy, green | 48.87 ± 4.37 ^b^ | 58.99 ± 5.41 ^a^ | 45.08 ± 2.76 ^b^ | 65.37 ± 1.36 ^a^ | 23.89 ± 2.25 ^a^ | 23.84 ± 1.33 ^a^ | 25.64 ± 1.47 ^a^ | 27.73 ± 0.85 ^a^ |
| Aldehydes | | | | | | | | | | | | | |
| 1 | Isopentanal | 590-86-3 | 652 | 665 | / | 0.13 ± 0.08 ^a^ | 0.08 ± 0.02 ^a^ | 0.10 ± 0.01 ^a^ | 0.10 ± 0.01 ^a^ | 0.15 ± 0.01 ^c^ | 0.28 ± 0.01 ^a^ | 0.23 ± 0.01 ^b^ | 0.15 ± 0.00 ^c^ |
| 2 | 2-Methylbutanal | 96-17-3 | 662 | 667 | / | 0.05 ± 0.01 ^a^ | 0.04 ± 0.00 ^a^ | 0.05 ± 0.01 ^a^ | 0.04 ± 0.00 ^a^ | 0.44 ± 0.02 ^a^ | 0.49 ± 0.02 ^a^ | 0.44 ± 0.02 ^a^ | 0.37 ± 0.01 ^b^ |
| 3 | Pentanal | 110-62-3 | 699 | 677 | Fruity, nutty, fermented | 0.30 ± 0.01 ^b^ | 0.09 ± 0.01^d^ | 0.38 ± 0.04 ^a^ | 0.21 ± 0.02 ^c^ | 0.30 ± 0.02 ^b^ | 0.28 ± 0.01 ^b^ | 0.35 ± 0.01 ^a^ | 0.35 ± 0.01 ^a^ |
| 4 | Hexanal | 66-25-1 | 800 | 825 | Grass, leafy, green | 1.84 ± 0.04 ^b^ | 2.47 ± 0.40 ^a^ | 2.98 ± 0.07 ^a^ | 2.69 ± 0.03 ^a^ | 2.35 ± 0.33 ^ab^ | 0.52 ± 0.04 ^c^ | 2.04 ± 0.13 ^b^ | 2.80 ± 0.04 ^a^ |
| 5 | Heptanal | 111-71-7 | 901 | 920 | Herbal, green, fatty, citrus | 0.38 ± 0.03 ^a^ | 0.40 ± 0.06 ^a^ | 0.37 ± 0.05 ^a^ | 0.36 ± 0.00 ^a^ | 0.68 ± 0.07 ^a^ | 0.47 ± 0.01 ^b^ | 0.35 ± 0.04 ^c^ | 0.46 ± 0.00 ^b^ |
| 6 | Benzaldehyde | 100-52-7 | 962 | 969 | Almond, cherry, sweet | 2.29 ± 0.55 ^c^ | 11.85 ± 1.41 ^ab^ | 9.75 ± 1.41 ^b^ | 12.93 ± 0.49 ^a^ | 3.83 ± 0.20 ^a^ | 2.40 ± 0.10 ^b^ | 2.29 ± 0.09 ^b^ | 2.34 ± 0.17 ^b^ |
| 7 | Octanal | 124-13-0 | 1003 | 1011 | / | 0.47 ± 0.12 ^a^ | 0.36 ± 0.08 ^a^ | 0.30 ± 0.08 ^a^ | 0.35 ± 0.03 ^a^ | 0.00 ± 0.00 | 0.00 ± 0.00 | 0.00 ± 0.00 | 0.00 ± 0.00 |
| 8 | Nonanal | 124-19-6 | 1104 | 1108 | Fatty, fresh, green | 1.75 ± 0.22 ^b^ | 2.48 ± 0.10 ^a^ | 2.45 ± 0.32 ^a^ | 2.64 ± 0.18 ^a^ | 0.00 ± 0.00 | 0.00 ± 0.00 | 0.00 ± 0.00 | 0.00 ± 0.00 |
| 9 | Decanal | 112-31-2 | 1206 | 1208 | Sweet, floral, waxy | 0.40 ± 0.03 ^a^ | 0.42 ± 0.06 ^a^ | 0.50 ± 0.09 ^a^ | 0.52 ± 0.02 ^a^ | 0.20 ± 0.00 ^c^ | 0.29 ± 0.03 ^b^ | 0.32 ± 0.02 ^b^ | 0.40 ± 0.02 ^a^ |
| 10 | β-Cyclocitral | 432-25-7 | 1220 | 1219 | Herbal, tobacco, sweet | 0.98 ± 0.10 ^ab^ | 0.79 ± 0.06 ^b^ | 0.78 ± 0.09 ^b^ | 1.07 ± 0.07 ^a^ | 0.53 ± 0.03 ^b^ | 0.59 ± 0.05 ^b^ | 0.49 ± 0.05 ^b^ | 0.81 ± 0.07 ^a^ |
| 11 | (*E*)-2-Decenal | 3913-81-3 | 1263 | 1263 | Green, mushroom, waxy | 0.24 ± 0.04 ^a^ | 0.20 ± 0.01 ^ab^ | 0.25 ± 0.01 ^a^ | 0.16 ± 0.01 ^b^ | 0.00 ± 0.00 ^b^ | 0.00 ± 0.00 ^b^ | 0.00 ± 0.00 ^b^ | 0.43 ± 0.01 ^a^ |
| Alkanes | | | | | | | | | | | | | |
| 1 | 3-Methylundecane | 1002-43-3 | 1170 | 1173 | / | 0.10 ± 0.02 ^b^ | 0.14 ± 0.01 ^a^ | 0.00 ± 0.00 ^c^ | 0.08 ± 0.01 ^b^ | 0.00 ± 0.00 | 0.00 ± 0.00 | 0.00 ± 0.00 | 0.00 ± 0.00 |
| 2 | Dodecane | 112-40-3 | 1200 | 1202 | Alkane | 1.03 ± 0.27 ^a^ | 1.19 ± 0.12 ^a^ | 0.93 ± 0.04 ^a^ | 1.12 ± 0.10 ^a^ | 0.35 ± 0.02 ^a^ | 0.24 ± 0.02 ^b^ | 0.25 ± 0.01 ^b^ | 0.38 ± 0.10 ^a^ |
| 3 | 3-Methyltridecane | 6418-41-3 | 1371 | 1371 | / | 0.22 ± 0.02 ^bc^ | 0.30 ± 0.03 ^a^ | 0.18 ± 0.01 ^c^ | 0.25 ± 0.00 ^b^ | 0.00 ± 0.00 | 0.00 ± 0.00 | 0.00 ± 0.00 | 0.00 ± 0.00 |
| 4 | Tetradecane | 629-59-4 | 1400 | 1400 | Waxy | 1.01 ± 0.42 ^b^ | 1.75 ± 0.10 ^a^ | 1.51 ± 0.02 ^ab^ | 1.58 ± 0.10 ^ab^ | 0.45 ± 0.01 ^a^ | 0.41 ± 0.01 ^b^ | 0.00 ± 0.00 ^c^ | 0.46 ± 0.02 ^a^ |
| 5 | Hexadecane | 544-76-3 | 1600 | 1600 | Alkane | 0.00 ± 0.00 ^b^ | 0.68 ± 0.18 ^a^ | 0.49 ± 0.01 ^a^ | 0.46 ± 0.02 ^a^ | 0.21 ± 0.02 ^b^ | 0.00 ± 0.00 ^c^ | 0.33 ± 0.03 ^a^ | 0.00 ± 0.00 ^c^ |
| Alkenes | | | | | | | | | | | | | |
| 1 | (*E*)-4,8-Dimethylnona-1,3,7-triene | 19945-61-0 | 1116 | 1120 | / | 1.69 ± 0.23 ^b^ | 2.58 ± 0.11 ^a^ | 2.77 ± 0.24 ^a^ | 2.68 ± 0.04 ^a^ | 0.65 ± 0.02 ^a^ | 0.55 ± 0.03 ^b^ | 0.28 ± 0.01 ^c^ | 0.58 ± 0.01 ^b^ |
| 2 | 2,6-Dimethyl-2,4,6-octatriene | 673-84-7 | 1131 | 1145 | / | 0.37 ± 0.06 ^a^ | 0.39 ± 0.03 ^a^ | 0.25 ± 0.06 ^b^ | 0.00 ± 0.00 ^c^ | 0.00 ± 0.00 | 0.00 ± 0.00 | 0.00 ± 0.00 | 0.00 ± 0.00 |
| 3 | Dihydrocurcumene | 1461-02-5 | 1448 | 1447 | / | 0.44 ± 0.15 ^a^ | 0.00 ± 0.00 ^b^ | 0.00 ± 0.00 | 0.00 ± 0.00 ^b^ | 0.20 ± 0.01 ^a^ | 0.00 ± 0.00 ^b^ | 0.00 ± 0.00 ^b^ | 0.00 ± 0.00 ^b^ |
| 4 | (*Z*)-β-Farnesene | 28973-97-9 | 1444 | 1458 | Citrus, sweet, woody | 11.64 ± 0.26 ^a^ | 9.61 ± 0.61 ^b^ | 8.08 ± 1.24 ^b^ | 11.69 ± 0.25 ^a^ | 3.89 ± 0.50 ^b^ | 0.00 ± 0.00 ^c^ | 4.01 ± 0.36 ^b^ | 5.48 ± 0.14 ^a^ |
| 5 | α-Curcumene | 644-30-4 | 1483 | 1482 | Herb | 0.53 ± 0.04 ^a^ | 0.26 ± 0.04 ^b^ | 0.16 ± 0.01 ^c^ | 0.32 ± 0.03 ^b^ | 0.26 ± 0.10 ^c^ | 0.53 ± 0.08 ^b^ | 0.98 ± 0.02 ^a^ | 0.16 ± 0.01 ^c^ |
| 6 | γ-Curcumene | 451-55-8 | 1480 | 1479 | / | 0.24 ± 0.00 ^a^ | 0.16 ± 0.03 ^b^ | 0.00 ± 0.00 ^c^ | 0.17 ± 0.01 ^b^ | 0.00 ± 0.00 | 0.00 ± 0.00 | 0.00 ± 0.00 | 0.00 ± 0.00 |
| 7 | trans-α-Bisabolene | 25532-79-0 | 1512 | 1502 | / | 0.26 ± 0.01 ^b^ | 0.45 ± 0.02 ^a^ | 0.50 ± 0.04 ^a^ | 0.23 ± 0.01 ^b^ | 0.23 ± 0.02 ^b^ | 0.19 ± 0.00 ^c^ | 0.20 ± 0.01 ^bc^ | 0.27 ± 0.00 ^a^ |
| 8 | β-Bisabolene | 495-61-4 | 1509 | 1508 | Balsamic | 5.83 ± 0.49 ^a^ | 0.00 ± 0.00 ^c^ | 0.00 ± 0.00 ^c^ | 4.75 ± 0.20 ^b^ | 1.96 ± 0.08 ^a^ | 1.60 ± 0.17 ^b^ | 2.01 ± 0.04 ^a^ | 2.09 ± 0.13 ^a^ |
| 9 | trans-γ-Bisabolene | 53585-13-0 | 1533 | 1599 | / | 0.82 ± 0.01 ^a^ | 0.63 ± 0.12 ^b^ | 0.72 ± 0.03 ^ab^ | 0.68 ± 0.04 ^ab^ | 0.00 ± 0.00 | 0.00 ± 0.00 | 0.00 ± 0.00 | 0.00 ± 0.00 |
| Aromatic compounds | | | | | | | | | | | | | |
| 1 | Toluene | 108-88-3 | 763 | 803 | Sweet, paint | 0.08 ± 0.00 ^b^ | 0.12 ± 0.01 ^a^ | 0.15 ± 0.02 ^a^ | 0.12 ± 0.00 ^a^ | 1.11 ± 0.02 ^a^ | 0.85 ± 0.04 ^c^ | 0.94 ± 0.04 ^b^ | 0.99 ± 0.01 ^b^ |
| 2 | 1,3-Dimethylbenzene | 108-38-3 | 866 | 887 | Plastic | 2.59 ± 0.11 ^a^ | 0.93 ± 0.03 ^c^ | 0.08 ± 0.01 ^d^ | 1.62 ± 0.05 ^b^ | 1.53 ± 0.15 ^a^ | 1.08 ± 0.06 ^b^ | 0.97 ± 0.12 ^b^ | 0.94 ± 0.05 ^b^ |
| 3 | Cinnamene | 100-42-5 | 893 | 909 | Balsam, plastic | 0.37 ± 0.14 ^a^ | 0.15 ± 0.01 ^b^ | 0.17 ± 0.05 ^ab^ | 0.15 ± 0.00 ^b^ | 0.18 ± 0.02 ^b^ | 0.16 ± 0.00 ^bc^ | 0.13 ± 0.01 ^c^ | 0.24 ± 0.02 ^a^ |
| 4 | 4-Methyl-1-pentanoylbenzene | 1671-77-8 | / | 1029 | / | 0.04 ± 0.01 ^a^ | 0.00 ± 0.00 ^b^ | 0.00 ± 0.00 ^b^ | 0.00 ± 0.00 ^b^ | 0.13 ± 0.02 ^a^ | 0.49 ± 0.63 ^a^ | 0.18 ± 0.00 ^a^ | 0.24 ± 0.03 ^a^ |
| 5 | Benzeneacetaldehyde | 122-78-1 | 1045 | 1048 | Floral, green, honey | 4.83 ± 0.32 ^c^ | 16.45 ± 0.54 ^a^ | 9.16 ± 0.64 ^b^ | 15.23 ± 0.32 ^a^ | 4.17 ± 0.24 ^c^ | 5.14 ± 0.39 ^b^ | 7.97 ± 0.23 ^a^ | 0.00 ± 0.00 ^d^ |
| 6 | 5-Methyl-1-phenyl-1-hexanone | 25552-17-4 | / | 1073 | / | 0.20 ± 0.05 ^c^ | 0.33 ± 0.02 ^b^ | 0.26 ± 0.00 ^bc^ | 0.46 ± 0.02 ^a^ | 0.39 ± 0.06 ^a^ | 0.00 ± 0.00 ^b^ | 0.00 ± 0.00 ^b^ | 0.42 ± 0.05 ^a^ |
| 7 | Benzyl nitrile | 140-29-4 | 1144 | 1142 | / | 8.49 ± 0.21 ^c^ | 11.51 ± 0.74 ^ab^ | 10.72 ± 0.71 ^b^ | 12.73 ± 0.18 ^a^ | 11.92 ± 0.18 ^c^ | 23.54 ± 0.85 ^ab^ | 21.89 ± 1.19 ^b^ | 26.93 ± 2.15 ^a^ |
| 8 | Propionylbenzene | 93-55-0 | 1176 | 1167 | Hawthorn, lilac | 0.15 ± 0.05 ^a^ | 0.18 ± 0.00 ^a^ | 0.16 ± 0.03 ^a^ | 0.16 ± 0.01 ^a^ | 0.26 ± 0.01 ^a^ | 0.00 ± 0.00 ^b^ | 0.22 ± 0.02 ^a^ | 0.25 ± 0.16 ^a^ |
| 9 | Indole | 120-72-9 | 1295 | 1294 | Floral, honey, jasmine | 75.73 ± 4.60 ^c^ | 99.45 ± 4.75 ^b^ | 114.28 ± 2.29 ^a^ | 110.65 ± 4.26 ^a^ | 73.47 ± 0.14 ^a^ | 76.65 ± 5.20 ^a^ | 73.44 ± 0.57 ^a^ | 74.59 ± 8.08 ^a^ |
| 10 | (2-Nitroethyl) benzene | 6125-24-2 | 1304 | 1298 | Flower, spice | 4.24 ± 0.38 ^c^ | 6.49 ± 0.45 ^b^ | 7.19 ± 0.49 ^b^ | 8.62 ± 0.43 ^a^ | 4.04 ± 0.12 ^a^ | 4.44 ± 0.17 ^a^ | 4.02 ± 0.00 ^a^ | 4.82 ± 0.89 ^a^ |
| Esters | | | | | | | | | | | | | |
| 1 | Ethyl acetate | 141-78-6 | 612 | 657 | / | 0.12 ± 0.01 ^a^ | 0.11 ± 0.01 ^a^ | 0.13 ± 0.02 ^a^ | 0.07 ± 0.00 ^b^ | 0.12 ± 0.01 ^b^ | 0.13 ± 0.02 ^ab^ | 0.10 ± 0.01 ^b^ | 0.15 ± 0.00 ^a^ |
| 2 | Methyl salicylate | 119-36-8 | 1192 | 1194 | Mint, wintergreen | 1.39 ± 0.20 ^c^ | 3.92 ± 0.26 ^a^ | 3.09 ± 0.22 ^b^ | 3.21 ± 0.11 ^b^ | 2.36 ± 0.25 ^c^ | 3.37 ± 0.37 ^b^ | 4.06 ± 0.03 ^a^ | 4.12 ± 0.16 ^a^ |
| 3 | (3*Z*)-3-Hexenyl 2-methylbutanoate | 53398-85-9 | 1234 | 1235 | Fresh, fruity, green | 0.42 ± 0.09 ^b^ | 0.47 ± 0.03 ^b^ | 0.54 ± 0.05 ^b^ | 0.75 ± 0.00 ^a^ | 0.00 ± 0.00 | 0.00 ± 0.00 | 0.00 ± 0.00 | 0.00 ± 0.00 |
| 4 | Isopentyl hexanoate | 2198-61-0 | 1252 | 1253 | Fruity, pine, apple | 0.07 ± 0.02 ^a^ | 0.07 ± 0.01 ^a^ | 0.06 ± 0.00 ^a^ | 0.06 ± 0.01 ^a^ | 0.00 ± 0.00 | 0.00 ± 0.00 | 0.00 ± 0.00 | 0.00 ± 0.00 |
| 5 | (*Z*)-3-Hexenyl hexanoate | 31501-11-8 | 1380 | 1383 | Fruity, grassy, tropical | 2.22 ± 0.38 ^b^ | 3.56 ± 0.02 ^a^ | 3.69 ± 0.58 ^a^ | 3.31 ± 0.09 ^a^ | 1.41 ± 0.24 ^a^ | 1.12 ± 0.12 ^a^ | 0.75 ± 0.01 ^b^ | 1.40 ± 0.03 ^a^ |
| 6 | Hexanoic acid, hexyl ester | 6378-65-0 | 1384 | 1388 | Fresh, fruity, vegetable | 0.30 ± 0.09 ^b^ | 0.59 ± 0.08 ^a^ | 0.34 ± 0.14 ^ab^ | 0.42 ± 0.03 ^ab^ | 0.13 ± 0.01 ^ab^ | 0.00 ± 0.00 ^c^ | 0.12 ± 0.01 ^b^ | 0.16 ± 0.01 ^a^ |
| 7 | β-Phenylethyl butyrate | 103-52-6 | 1447 | 1395 | / | 0.19 ± 0.05 ^b^ | 0.25 ± 0.03 ^b^ | 0.60 ± 0.13 ^a^ | 0.23 ± 0.01 ^b^ | 0.12 ± 0.00 ^c^ | 0.64 ± 0.05 ^a^ | 0.47 ± 0.02 ^b^ | 0.59 ± 0.02 ^a^ |
| 8 | Decanoic acid, ethyl ester | 110-38-3 | 1396 | 1398 | Fruity, sweet, oily | 15.13 ± 1.38 ^c^ | 20.91 ± 1.35 ^b^ | 19.69 ± 0.43 ^b^ | 25.55 ± 0.71 ^a^ | 11.56 ± 0.15 ^c^ | 19.21 ± 0.44 ^b^ | 19.38 ± 0.92 ^b^ | 21.41 ± 0.33 ^a^ |
| 9 | 2,4-Ditert-butylphenyl 5-hydroxypentanoate | 166273-38-7 | / | 1515 | / | 0.12 ± 0.01 ^b^ | 0.26 ± 0.03 ^a^ | 0.13 ± 0.01 ^b^ | 0.25 ± 0.02 ^a^ | 0.16 ± 0.02 ^a^ | 0.10 ± 0.02 ^b^ | 0.10 ± 0.01 ^b^ | 0.16 ± 0.01 ^a^ |
| 10 | Methyl jasmonate | 1211-29-6 | 1638 | 1752 | Floral, jasmine, fresh | 0.00 ± 0.00 ^c^ | 0.23 ± 0.06 ^b^ | 0.39 ± 0.05 ^a^ | 0.25 ± 0.03 ^b^ | 0.00 ± 0.00 | 0.00 ± 0.00 | 0.00 ± 0.00 | 0.00 ± 0.00 |
| 11 | 2-Ethylhexyl salicylate | 118-60-5 | 1811 | 1877 | / | 0.06 ± 0.01 ^a^ | 0.11 ± 0.05 ^a^ | 0.10 ± 0.02 ^a^ | 0.06 ± 0.00 ^a^ | 0.05 ± 0.01 ^b^ | 0.08 ± 0.01 ^a^ | 0.03 ± 0.01 ^b^ | 0.05 ± 0.00 ^b^ |
| 12 | Jasmine lactone | 25524-95-2 | 1518 | 1491 | Coconut, creamy, jasmine | 6.11 ± 0.56 ^b^ | 7.72 ± 0.92 ^ab^ | 4.98 ± 0.78 ^b^ | 8.45 ± 0.15 ^a^ | 7.96 ± 0.09 ^a^ | 6.06 ± 0.47 ^b^ | 4.40 ± 0.38 ^c^ | 7.98 ± 0.07 ^a^ |
| Ketones | | | | | | | | | | | | | |
| 1 | 1-Penten-3-one | 1629-58-9 | 681 | 674 | / | 0.12 ± 0.01 ^a^ | 0.07 ± 0.01 ^b^ | 0.07 ± 0.00 ^b^ | 0.12 ± 0.01 ^a^ | 0.28 ± 0.01 ^ab^ | 0.33 ± 0.06 ^a^ | 0.21 ± 0.01 ^b^ | 0.32 ± 0.01 ^a^ |
| 2 | 2-Heptanone | 110-43-0 | 891 | 911 | Sweet, fruity, spicy, woody | 0.07 ± 0.01 ^b^ | 0.00 ± 0.00 ^c^ | 0.10 ± 0.02 ^a^ | 0.00 ± 0.00 ^c^ | 0.33 ± 0.01 ^a^ | 0.29 ± 0.05 ^a^ | 0.27 ± 0.03 ^a^ | 0.32 ± 0.01 ^a^ |
| 3 | 6-Methyl-5-heptene-2-one | 110-93-0 | 986 | 997 | Mushroom, pepper, rubber | 4.79 ± 0.23 ^b^ | 7.31 ± 0.62 ^a^ | 6.84 ± 0.61 ^a^ | 5.49 ± 0.13 ^b^ | 3.97 ± 0.17 ^a^ | 3.79 ± 0.31 ^a^ | 4.08 ± 0.07 ^a^ | 3.93 ± 0.46 ^a^ |
| 4 | 2,2,6-Trimethylcyclohexanone | 2408-37-9 | 1036 | 1038 | Cistus, honey, pungent | 0.29 ± 0.05 ^a^ | 0.19 ± 0.02 ^b^ | 0.20 ± 0.02 ^b^ | 0.29 ± 0.01 ^a^ | 0.30 ± 0.04 ^a^ | 0.32 ± 0.01 ^a^ | 0.25 ± 0.01 ^b^ | 0.32 ± 0.01 ^a^ |
| 5 | (3*E*)-5-Ethyl-6-methyl-3-hepten-2-one | 57283-79-1 | 1144 | 1149 | / | 0.14 ± 0.02 ^a^ | 0.00 ± 0.00 ^b^ | 0.12 ± 0.02 ^a^ | 0.00 ± 0.00 ^b^ | 0.11 ± 0.00 ^b^ | 0.00 ± 0.00 ^c^ | 0.13 ± 0.01 ^a^ | 0.00 ± 0.00 ^c^ |
| 6 | α-Ionone | 127-41-3 | 1426 | 1427 | Fruity, violet, woody | 0.22 ± 0.00 ^b^ | 0.26 ± 0.08 ^b^ | 0.28 ± 0.07 ^b^ | 0.52 ± 0.02 ^a^ | 0.18 ± 0.00 ^c^ | 0.44 ± 0.03 ^b^ | 0.15 ± 0.01 ^c^ | 0.63 ± 0.04 ^a^ |
| 7 | (*E*)-β-Ionone | 79-77-6 | 1486 | 1485 | Floral, violet, powdery | 1.75 ± 0.11 ^b^ | 2.50 ± 0.28 ^a^ | 2.54 ± 0.39 ^a^ | 3.18 ± 0.22 ^a^ | 1.60 ± 0.31 ^c^ | 3.72 ± 0.21 ^b^ | 1.54 ± 0.05 ^c^ | 4.37 ± 0.24 ^a^ |
| Heterocyclic compounds | | | | | | | | | | | | | |
| 1 | 2-Ethylfuran | 3208-16-0 | 703 | 678 | Sweet, malty, earthy | 0.12 ± 0.03 ^a^ | 0.10 ± 0.00 ^a^ | 0.14 ± 0.01 ^a^ | 0.10 ± 0.01 ^a^ | 0.16 ± 0.03 ^a^ | 0.17 ± 0.01 ^a^ | 0.17 ± 0.01 ^a^ | 0.15 ± 0.01 ^a^ |
| 2 | 2-Pentylfuran | 3777-69-3 | 993 | 1000 | Butter, green bean | 0.65 ± 0.04 ^a^ | 0.64 ± 0.07 ^a^ | 0.74 ± 0.05 ^a^ | 0.77 ± 0.03 ^a^ | 0.00 ± 0.00 | 0.00 ± 0.00 | 0.00 ± 0.00 | 0.00 ± 0.00 |

LST: light-scented *Tieguanyin*; SST: strong-scented *Tieguanyin*; PW: pure water; MSW: mountain spring water; MW: mineral water; NW: natural water. Data are means (± SD) of three replicates. The relative concentrations of volatile compounds were determined by comparing the total ion flow response of the compounds in mass spectra with that of ethyl decanoate. ^a,b,c,d^ Different letters in the same row indicate significant differences between mean values (*p* < 0.05).


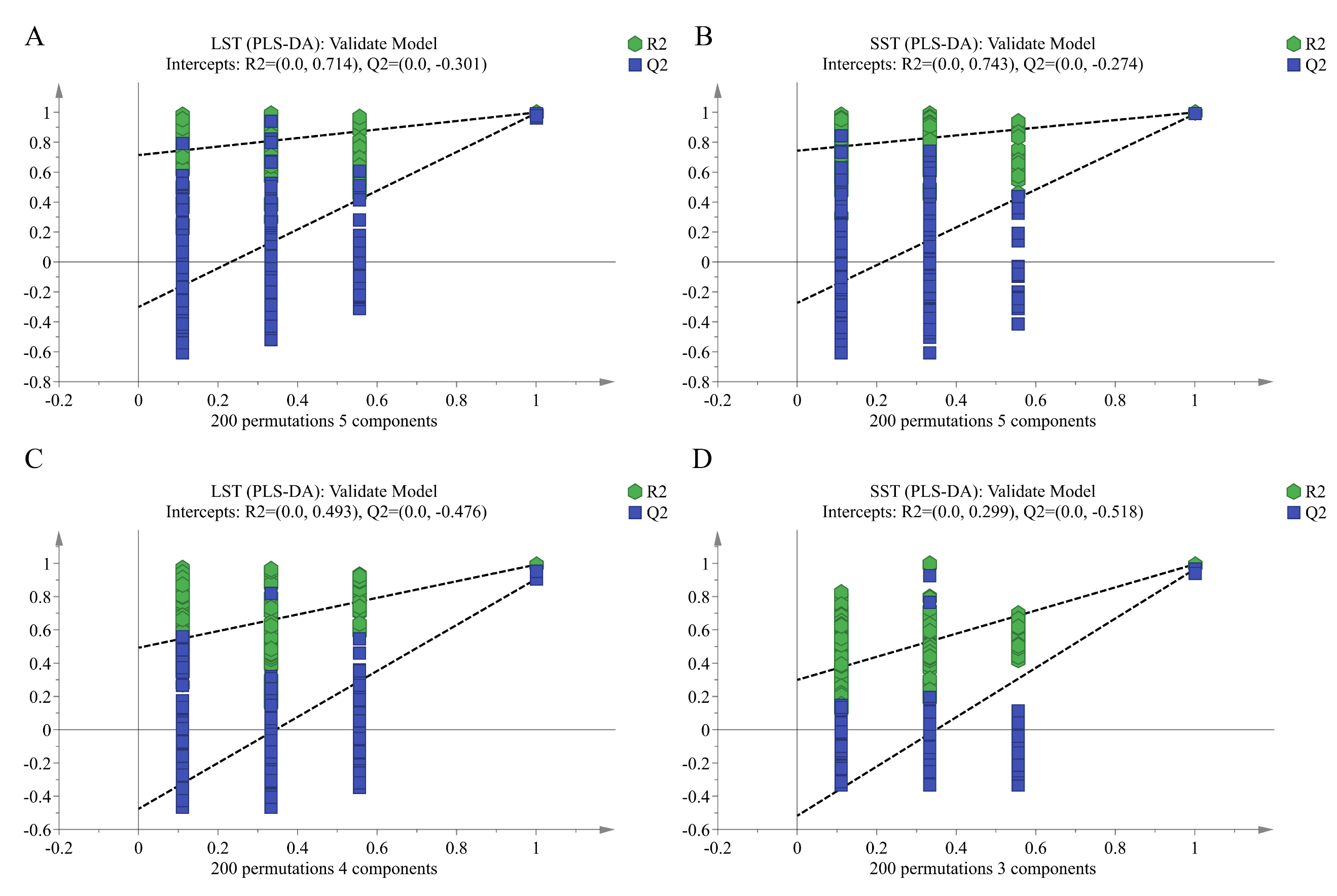


Fig. S1. The permutation test results of the partial least squares-discrimination analysis (PLS-DA).

A & B: The non-volatile compounds in LST & SST; C & D: The volatile compounds in LST & SST. LST: light-scented *Tieguanyin*; SST: strong-scented *Tieguanyin*.
